# Multi-Mode Hand Gesture-Based VR Locomotion Technique for Intuitive Telemanipulation Viewpoint Control in Tightly Arranged Logistic Environments

**DOI:** 10.3390/s25041181

**Published:** 2025-02-14

**Authors:** Jaehoon Jeong, Haegyeom Choi, Donghun Lee

**Affiliations:** Mechanical Engineering Department, Soongsil University, Seoul 06978, Republic of Korea; wogns1218@soongsil.ac.kr (J.J.); choihg@soongsil.ac.kr (H.C.)

**Keywords:** telemanipulation, VR locomotion, logistics robots, hand gesture recognition, multi-layer perceptron

## Abstract

Telemanipulation-based object-side picking with a suction gripper often faces challenges such as occlusion of the target object or the gripper and the need for precise alignment between the suction cup and the object’s surface. These issues can significantly affect task success rates in logistics environments. To address these problems, this study proposes a multi-mode hand gesture-based virtual reality (VR) locomotion method to enable intuitive and precise viewpoint control. The system utilizes a head-mounted display (HMD) camera to capture hand skeleton data, which a multi-layer perceptron (MLP) model processes. The model classifies gestures into three modes: translation, rotation, and fixed, corresponding to fist, pointing, and unknown gestures, respectively. Translation mode moves the viewpoint based on the wrist’s displacement, rotation mode adjusts the viewpoint’s angle based on the wrist’s angular displacement, and fixed mode stabilizes the viewpoint when gestures are ambiguous. A dataset of 4312 frames was used for training and validation, with 666 frames for testing. The MLP model achieved a classification accuracy of 98.4%, with precision, recall, and F1-score exceeding 0.98. These results demonstrate the system’s ability to address the challenges of telemanipulation tasks by enabling accurate gesture recognition and seamless mode transitions.

## 1. Introduction

**Figure 1:**
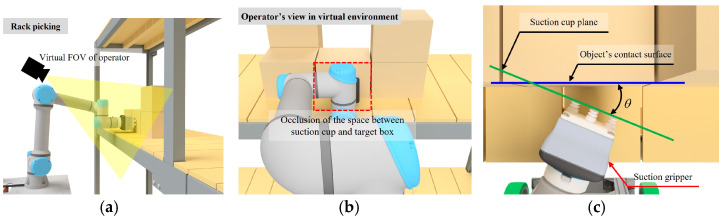
Challenges in telemanipulation-based object handling in logistics environments. (**a**) Illustrates telemanipulation-based object-side picking with a suction gripper in a logistics environment. (**b**) Illustrates the occlusion of the target object or gripper, which hinders task execution. (**c**) Between the suction cup and the object’s contact surface, larger θ values reduce the chances of successful picking.

With the advancements in deep learning and robotics technologies, the seamless integration of production and logistics processes has become a critical requirement in intelligent factories and manufacturing automation. In particular, achieving a smooth connection between production lines and warehouses is essential for implementing Just-in-Time (JIT) production systems and highly efficient material supply systems. However, it is challenging to perform automated pick-and-place tasks in complex working environments with densely arranged objects or rapidly changing object placements. Numerous studies have addressed these issues [1,2,3,4,5]. Among them, systems combining remote control and VR technologies, such as the method proposed by Galarza et al. (2023) [3], have garnered significant attention. Nevertheless, these systems face challenges when the target object or gripper is occluded, making task execution difficult. This issue becomes even more pronounced in telemanipulation-based object-side picking with a suction gripper, as illustrated in Figure 1. As shown in Figure 1c, the success of pick-and-place tasks involving a mobile manipulator and a suction gripper critically depends on the precise positional relationship between the suction cup and the contact surface of the object. To enable precise control of the mobile manipulator’s end effector, operators must have a clear and stable viewpoint that allows for accurate observation of the target object. Unlike conventional VR locomotion techniques designed for navigating large virtual environments, the primary challenge in this study lies in managing viewpoint transitions within a constrained workspace to facilitate precise telemanipulation tasks. Therefore, an effective viewpoint control mechanism is necessary to ensure seamless and intuitive adjustments without relying on additional physical movement or external tracking devices.

Boletsis (2017) [6] classified VR locomotion techniques into four categories—roomscale-based, controller-based, teleportation-based, and motion-based—based on interaction type (physical or artificial), VR motion type (continuous or non-continuous), and VR interaction type (open or limited). The three methods, excluding the Motion-based approach, exhibit limitations in telemanipulating mobile manipulators within logistics environments.

- Roomscale-based method: The roomscale-based method, which reflects the user’s physical movement in the virtual environment, offers high immersion and intuitive interaction. Bozgeyikli et al. (2019) [7] implemented this approach using tracking technology. Unlike other locomotion techniques, this method minimizes cybersickness because the user’s real-world movement directly corresponds to the visual motion in the virtual environment, preventing sensory conflict between the visual and vestibular systems. As a result, users experience a more natural sense of presence with reduced discomfort. However, this method is constrained by the physical size of the real-world environment, limiting user movement to within the boundaries of the actual space. For instance, in confined spaces or environments with many obstacles, user movement is restricted, making it challenging to secure visibility for the telemanipulation of a mobile manipulator. Consequently, this method is unsuitable for logistics environments requiring expansive workspaces.
- Teleportation-based method: The teleportation-based method [8,9,10] allows users to move to a selected location instantly without being limited by the size of the physical space. Bozgeyikli et al. (2016) [8] implemented this approach using Point and Teleport technology, enabling fast and intuitive position changes. However, this method lacks continuity in movement, and the sudden shift in perspective can cause users to lose spatial awareness. Furthermore, logistics environments often require tasks at varying heights and complex operations. Still, most Teleportation-based methods only support movement at fixed heights, making it challenging to maintain the necessary visibility. These limitations make it difficult to perform complex tasks effectively in logistics settings.
- Controller-based method: The controller-based method uses a joystick or sensors to enable users to control their viewpoint continuously in VR without physical movement. Englmeier et al. (2020) [11] proposed a technique utilizing a sphere-shaped device that allows users to control their viewpoint through rotation and tilting, demonstrating that precise and intuitive control is achievable in virtual environments. However, this approach requires additional devices and sensors, such as a joystick or Inertial Measurement Unit (IMU), beyond an HMD, leading to increased initial costs and additional expenses for maintenance.

The motion-based method [12,13,14,15,16,17,18] supports continuous movement within virtual environments and, in some cases, can be implemented using only an HMD. However, not all motion-based methods are free from physical space constraints. While techniques such as walk-in-place and gesture-based movement operate in confined spaces, others—like Redirected Walking—still require a minimum physical area to function properly. Additionally, motion-based methods vary in their hardware requirements. Some, such as gesture-based approaches, can be implemented using only an HMD’s built-in tracking system. However, others, like arm swinging, typically require external controllers or sensors to ensure accurate tracking. Even with inside-out tracking, maintaining hand visibility within the HMD’s field of view can be challenging, especially for full-arm motion techniques. While certain motion-based methods reduce hardware dependencies, their practical implementation depends on tracking capabilities, physical constraints, and the requirements of the task.

- Walk-in-place method: The walk-in-place method detects the user’s stepping motion in place to generate a velocity vector and translates it into movement within the virtual environment. Lee et al. (2018) [12] proposed a method that detects stepping motions based on HMD posture data and connects them to virtual navigation. While this approach provides users with a natural movement experience, it requires continuous stepping, which can be physically exhausting. Additionally, when combined with the telemanipulation of a mobile manipulator, unintended hand movements may occur, reducing the precision of the telemanipulation.
- Arm swinging method: The arm swinging method uses the user’s arm movements as input to implement navigation. Pai and Kunze (2017) [14] proposed a technique that detects arm motions to control movement within a virtual environment. However, this method requires continuous use of both arms, making it unsuitable for telemanipulation of a mobile manipulator, where precise hand control is needed. Additionally, the repeated arm swinging motion can be physically tiring, making it difficult for users to maintain over long periods.
- Gesture-based method: The gesture-based method uses the user’s body movements to enable navigation and interaction within virtual environments. This includes hand gestures and head, arm, and full-body motions. Users can easily adjust their perspective or navigate the virtual environment with simple actions, reducing physical strain compared to other methods.

The gesture-based method utilizes various body movements, with hand gestures being the most widely used primary input technique. As a result, hand gesture recognition (HGR) has emerged as a core technology for gesture-based methods. HGR refers to accurately identifying user hand movements and linking them to tasks such as navigation or viewpoint control in virtual environments. It can be divided into sensor-based and computer vision-based approaches. The sensor-based approach collects data using devices such as IMUs [19,20,21], electromyography (EMG) sensors [22,23], and flex sensors [24]. While this method offers high precision, it requires additional hardware, such as microcontrollers, for data processing and transmission. On the other hand, the computer vision-based approach does not require sensors to be attached to the user’s body, offering greater convenience. In VR, this approach leverages cameras mounted on the HMD, allowing for implementation without additional hardware. Oudah et al. (2020) [25] categorized computer vision-based HGR technologies into seven types based on recognition methods. Among these, skeleton recognition [26,27,28] analyzes hand movements using skeletal structure, enhancing the detection of complex features. A notable example is MediaPipe, proposed by Lugaresi et al. (2019) [28], enabling real-time hand skeleton data extraction using a single camera image.

To address these limitations, this study proposes a method that eliminates workspace constraints while using only an HMD for tracking. While previous studies, such as Kirihata and Ishikawa (2024) [18], explored single-hand gesture-based locomotion, their approach was based on teleportation, which allows discrete movement transitions rather than continuous viewpoint control. In contrast, the proposed method enables continuous viewpoint manipulation using a single hand, allowing operators to adjust their position and orientation smoothly without predefined teleportation points. By extracting hand skeleton data through the HMD’s built-in cameras and processing it with an MLP model, the system classifies gestures in real time and applies transformations in translation, rotation, and fixed modes. This approach allows for more precise viewpoint adjustments in telemanipulation tasks, where operators need fluid motion rather than stepwise relocation. The ability to control the viewpoint with one hand also ensures that the other hand remains available for object manipulation, making this method more suitable for logistics and industrial applications.

## 2. Materials and Methods

**Figure 2:**
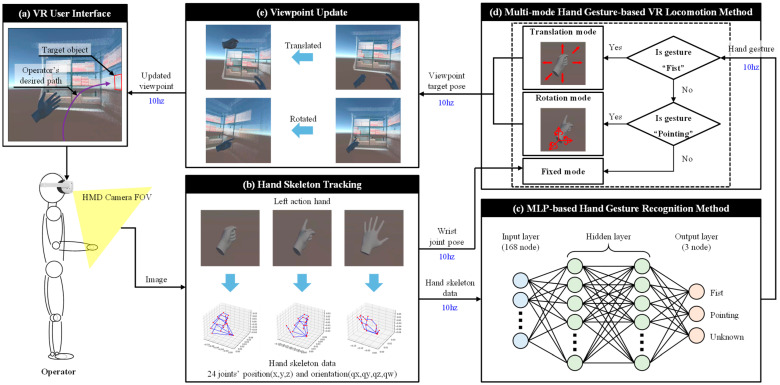
Framework of the proposed hand gesture-based VR locomotion technique. (**a**) VR user interface: real-time visualization of the current viewpoint; (**b**) hand skeleton tracking: real-time extraction of 24 joint positions and orientations using HMD cameras; (**c**) MLP-based hand gesture recognition method: classification of hand gestures (fist, pointing, and unknown) for VR locomotion; (**d**) multi-mode hand gesture-based VR locomotion method: integration of translation, rotation, and fixed modes based on hand gestures; and (**e**) viewpoint update: gesture-controlled updates of the viewpoint according to the user’s actions in the virtual environment.

This section explains the structure and operation of the VR locomotion technique designed to enable operators to intuitively control their viewpoint in a virtual environment for logistics tasks. Figure 2 illustrates the overall structure of the proposed method, encompassing the processes of real-time hand skeleton data extraction and hand gesture classification and linking these to viewpoint translation and rotation within the virtual environment.

The operator visually navigates the virtual environment using an HMD and controls the viewpoint based on hand movements. The HMD’s camera captures real-time images of the operator’s hand and extracts 3D position and orientation data for 24 joints (skeleton data). These data are input into an MLP model, classifying the gestures into three categories: fist, pointing, and unknown. These gestures control viewpoint translation and rotation within the virtual environment. Each gesture is mapped to one of three actions: translation, rotation, or fixed, enabling the operator to manipulate the viewpoint without being constrained by physical space.

### 2.1. VR User Interface

**Figure 3:**
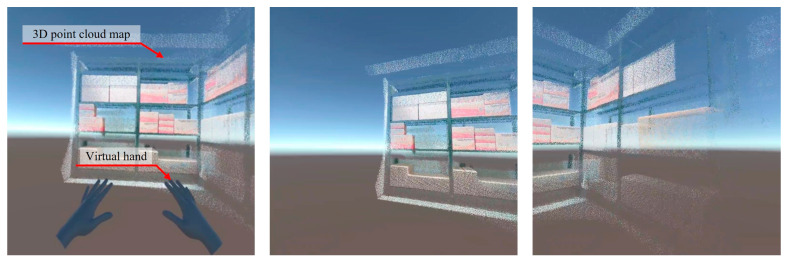
Illustrations of the VR user interface featuring a virtual hand and 3D point cloud map.

The user interface for the proposed VR locomotion technique is designed to connect the operator’s physical movements with the virtual environment while providing environmental information about the robot. As shown in Figure 3, the interface consists of a virtual hand, which virtualizes the operator’s real hand, and a 3D point cloud map generated using RTAB-Map [29].

The virtual hand is generated based on hand skeleton data tracked by the HMD camera, reflecting the skeleton data in real time. The operator’s hand movements are accurately represented in the virtual environment through the virtual hand, allowing the operator to understand the relationship between gestures and viewpoint control clearly.

The 3D point cloud map is a pre-generated dataset created using RTAB-Map. RTAB-Map utilizes sensors like RGB-D cameras to represent the workspace’s structure and objects’ positions as three-dimensional data. This map provides detailed information about the workspace within the virtual environment, allowing the operator to understand their current position and surroundings accurately. Compared to 2D images, 3D data offer a clear representation of depth and spatial structure, making it easier to identify objects’ relative positions and sizes. Additionally, 3D data include comprehensive spatial information, enabling the operator to explore the workspace from different angles and reducing information loss during viewpoint transitions.

### 2.2. Hand Skeleton Tracking

To reflect the operator’s hand movement data in the virtual environment in real time, this study utilizes the Oculus Integration SDK [30] to extract hand skeleton data. It implements a virtual hand based on these data. The extracted skeleton data consist of 24 joints, including the wrist, with each joint represented by a 3D position (x, y, z) and rotation (quaternion: qx, qy, qz, qw).

The camera embedded in the HMD captures real-time images of the operator’s hand, and the extracted data consist of the following joints:

- Forearm: the forearm joint, which connects to the wrist.
- Wrist: the wrist joint, connecting to the finger joints.
- Thumb: it consists of five joints—trapezium, metacarpal, proximal, distal, and tip.
- Index, Middle, Ring: each finger comprises four joints—proximal, intermediate, distal, and tip.
- Pinky: it includes five joints—metacarpal, proximal, intermediate, distal, and tip.

These data are used to map the operator’s hand movements to the virtual hand within the virtual environment and serves as the core input for the hand gesture-based locomotion technique proposed in this study.

### 2.3. MLP-Based Hand Gesture Recognition

This study designed the MLP model based on the previously described hand skeleton data to classify the operator’s hand gestures into three categories: fist, pointing, and unknown. This process establishes a foundation for intuitively controlling viewpoint translation and rotation within the virtual environment using hand gestures. Notably, the skeleton data extracted from the Oculus Quest 2 (Meta Platforms, Menlo Park, CA, USA), which include the joint data, are converted into a relative coordinate system centered on the wrist joint. This ensures data consistency, allowing gestures to be recognized uniformly regardless of the angle at which the operator raises their arm.

The dataset consists of 4312 frames, which were extracted from a continuous video where a person repeatedly performed random hand gestures. These frames were divided into a training set (80%) and a validation set (20%). The training set was used to optimize the model’s weights, while the validation set was used to monitor overfitting and determine the optimal stopping point during training. Since the hand gesture recognition model classifies gestures on a frame-by-frame basis, each frame was manually labeled according to the gesture being performed at that moment. The segmentation process involved reviewing the continuous video and assigning a gesture label to every individual frame, ensuring that each frame accurately represented a single hand posture. To maintain consistency, no overlapping frames were included, and ambiguous transitions between gestures were carefully excluded. The dataset composition used in this study is summarized in Table 1.

The MLP model consists of an input layer, two hidden layers, and an output layer. The input layer comprises 168 nodes, combining the skeleton data. The subsequent two hidden layers contain 128 and 64 nodes, respectively. The ReLU (Rectified Linear Unit) activation function is applied in the hidden layers, which helps introduce non-linearity by ensuring that only positive input values are passed forward. In contrast, negative values are replaced with zero. This enables the model to learn complex patterns and relationships within the data. Finally, the output layer uses a SoftMax function to calculate probability values for the three gesture categories: fist, pointing, and unknown.

The loss function used for training is categorical cross-entropy, widely utilized for multi-class classification problems. The Adam optimizer was chosen as the optimization algorithm. The batch size was set to 32 to enhance training efficiency, and the model was trained over 100 epochs. During training, the loss and accuracy of the training and validation sets were monitored at each epoch. To prevent overfitting, the early stopping technique was applied, terminating the training process early if no further improvement in performance was observed on the validation set.

After training, the model achieved approximately 99.5% accuracy on the training data and about 97.7% accuracy on the validation set. This demonstrates that the MLP model, utilizing skeleton data transformed into a relative coordinate system, classifies hand gestures with high accuracy. Subsequently, the three gestures are mapped to translation mode, rotation mode, and fixed mode within the virtual environment, serving as the input for the VR locomotion technique proposed in this study.

### 2.4. Multi-Mode Hand Gesture-Based VR Locomotion Method

Building on the MLP-based hand gesture recognition results, this section introduces a VR locomotion technique that integrates three modes—translation, rotation, and fixed—within the virtual environment. In this method, a hand gesture classified as fist activates the translation mode, enabling the operator to move the viewpoint by displacing the wrist. A gesture recognized as pointing enables the rotation mode, allowing the operator to rotate the viewpoint by adjusting the wrist’s angular displacement. The pointing gesture was chosen for this mode because the direction of the extended index finger provides an intuitive reference for rotational movement, making it easier for operators to control the rotation axis based on natural wrist motion. Lastly, for gestures classified as unknown, the fixed mode is triggered, preventing unintended viewpoint movements and ensuring seamless transitions between modes based on the operator’s hand movements.

**Figure 4:**
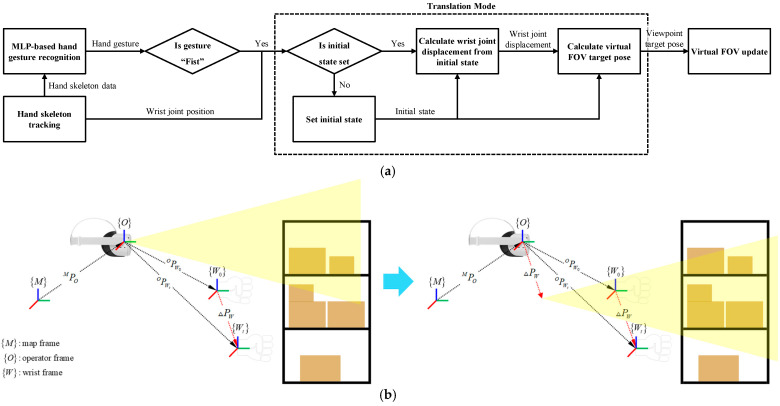
Illustration of the overall process of translation mode in the proposed VR locomotion method. (**a**) The flowchart depicts the process of translation mode. (**b**) Schematic representation of the translation mode operation.

Figure 4 illustrates the overall process of the translation mode. When a hand gesture is recognized as a fist, the system checks whether the wrist joint’s initial state has been set. If the initial state is not yet defined, the current wrist position is recorded as the initial state. Subsequently, if the hand gesture continues to be recognized as a fist, the displacement $\Delta P_{W}$ from the initial position PW0O to the current position PWtO is calculated as shown in Equation (1). The current viewpoint position PVtO is then updated to the position obtained by moving the initial viewpoint position PV0O by $\Delta P_{W}$. This process is expressed in Equation (2).

**Figure 5:**
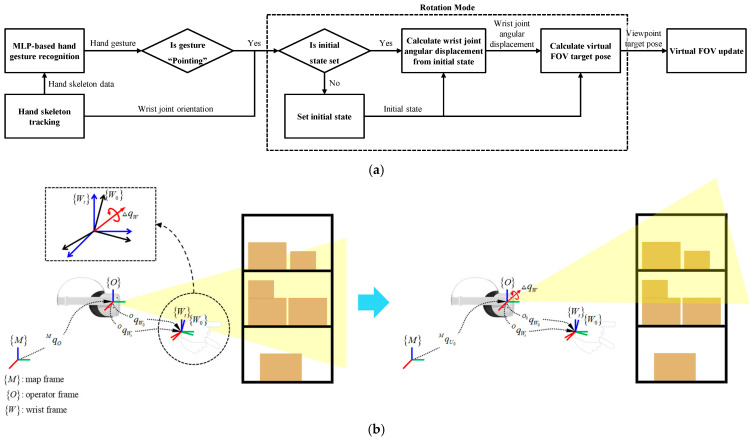
Illustration of the overall process of rotation mode in the proposed VR locomotion method. (**a**) The flowchart depicts the process of rotation mode. (**b**) Schematic representation of the rotation mode operation.

(1) ΔPW=PWtO−PW0O,

(2) PVtO=PV0O+ΔPW,

Figure 5 illustrates the overall process of the rotation mode. When a hand gesture is recognized as pointing, the system first checks whether the initial orientation of the wrist joint has been set. If the initial orientation is not yet defined, the current wrist orientation (quaternion) is recorded as the initial orientation. Subsequently, if the hand gesture continues to be recognized as pointing, the angular displacement $\Delta q_{W}$ from the initial orientation qW0O to the current orientation qWtO is calculated as shown in Equation (3). The initial viewpoint orientation qV0O is then rotated by $\Delta q_{W}$ to update the current viewpoint orientation qVtO. The formula for updating the current orientation is provided in Equation (4).

(3) ΔqW=qW0O−1qWtO,

(4) qVtO=ΔqWqV0O

Finally, when a hand gesture is classified as an unknown motion, the fixed mode is applied, keeping the current viewpoint stationary. This prevents unintended viewpoint movements caused by accidental hand gestures during operations and helps maintain a stable visual composition. The three modes can transition seamlessly between each other, allowing the operator to navigate, rotate, or fix their viewpoint within the virtual environment using only fist and pointing gestures.

## 3. Results

### 3.1. Performance of MLP-Based Hand Gesture Recognition

**Figure 6:**
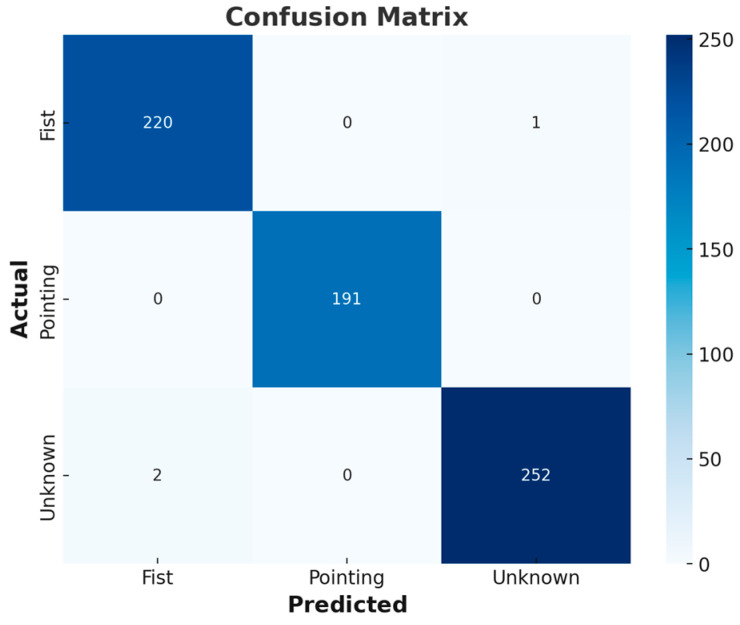
Confusion matrix of the prediction results for the test dataset.

In this study, a test dataset consisting of 666 sequentially collected data points was used to evaluate the performance of hand gesture recognition. The dataset was obtained from a single participant who randomly repeated hand gestures in a controlled environment. The participant was not given any specific task but was instructed to perform gestures naturally to ensure variability in execution. As shown in Figure 6, a confusion matrix was used to compare the model’s predictions with the actual values, visually representing correctly classified and misclassified cases for each class.

Subsequently, four performance metrics were measured: accuracy, precision, recall, and F1-score. Accuracy represents the proportion of test data the model correctly predicted, calculated as 0.984 in this study, meaning that approximately 98.4% of the 666 data points were correctly classified. Precision indicates the proportion of true positive cases among those predicted as positive, and a precision score of 0.985 signifies that 98.5% of the predicted hand gestures were actual gestures. Recall, which measures the proportion of true positive cases identified by the model out of all actual positive cases, was recorded as 0.984, confirming that the model successfully recognized most hand gestures without significant omission. Finally, the F1-score, which reflects the balance between precision and recall, was measured at 0.985, demonstrating consistently high performance across both metrics.

The confusion matrix analysis results and the four performance metrics demonstrate that this study’s MLP-based model achieves high accuracy and reliable predictive capability in the hand gesture recognition task.

### 3.2. Target Object Observation with Multi-Mode Hand Gesture-Based VR Locomotion Method

**Figure 7:**
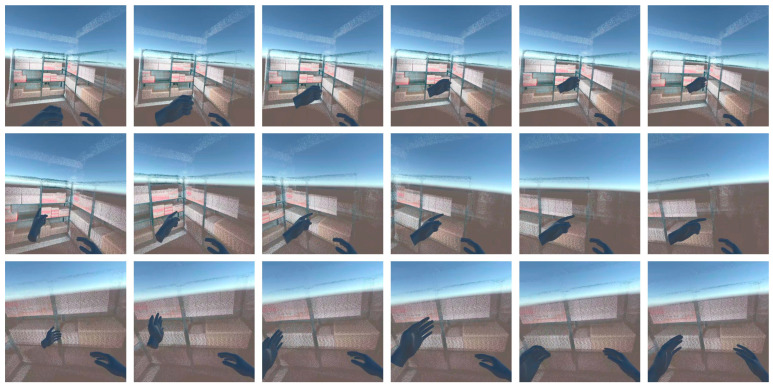
The proposed multi-mode hand gesture-based VR locomotion method represents the target object observation process. (**top**) Translation mode is activated by the fist gesture, moving the viewpoint closer to the target object. (**middle**) Rotation mode is triggered by the pointing gesture, adjusting the viewpoint angle to align with the target object. (**bottom**) The unknown gesture maintains the fixed mode, stabilizing the viewpoint near the target object. For a more detailed demonstration, please refer to the Supplementary Materials Video S1.

In this section, a task was conducted to observe a target object using the hand gesture-based VR locomotion method with the three modes, as shown in Figure 7. Each gesture triggered a specific viewpoint movement mode, and the proposed method was evaluated for its proper functionality across the three modes. The experimental results showed that each gesture accurately facilitated viewpoint adjustments, enabling the operator to observe the target object effectively.

The fist gesture was set to activate the translation mode, which is designed to move the viewpoint based on the displacement of the wrist joint. After the fist gesture was input during the experiment, the viewpoint shifted according to the wrist’s displacement. This allowed the operator to observe the object from closer or farther distances, with the viewpoint movement accurately responding to the displacement specified by the operator. This transformation process was implemented using Equations (1) and (2) from Section 2.4. Specifically, the system captured the initial position of the wrist joint upon detecting the fist gesture and then computed the displacement relative to the initial position. The resulting transformation matrix was applied to the viewpoint position, ensuring that the movement was precisely aligned with the operator’s hand motion.

The pointing gesture corresponds to the rotation mode, which is designed to rotate the viewpoint based on changes in the angle of the wrist joint. After inputting the pointing gesture, the viewpoint rotated according to the angular displacement of the wrist joint. In the experiment, the operator could observe the target object from their desired angle, with the viewpoint rotation precisely matching the wrist’s movement. This process was governed by Equations (3) and (4), where the initial wrist orientation was recorded upon gesture detection, and the angular displacement was calculated to adjust the viewpoint rotation. The system applied this transformation in real time, ensuring smooth and natural rotation control.

The unknown gesture was assigned to the fixed mode to maintain the current viewpoint without any changes. During the experiment, the system kept the viewpoint stationary when an unknown gesture was input. The fixed mode functioned as a standby state, holding the viewpoint steady while awaiting movement input from the other two modes.

## 4. Discussion

This study proposed a multi-mode hand gesture-based VR locomotion method that enables viewpoint control without requiring external sensors or controllers beyond the HMD. The proposed system consists of translation, rotation, and fixed modes, where real-time gesture recognition is used to adjust the viewpoint accordingly. The ability to operate with a single hand allows the operator to manipulate objects with the other hand, which is particularly beneficial in telemanipulation environments. Experimental results demonstrated that the MLP-based gesture classification model achieved a high accuracy of 98.4%, ensuring stable transitions between movement modes.

The proposed approach differs from existing gesture-based locomotion techniques in two key aspects. First, unlike the teleportation method used in Kirihata and Ishikawa (2024) [16], this study enables continuous viewpoint control. While teleportation offers rapid movement, it may cause spatial disorientation due to the abrupt relocation of the operator’s virtual position. In contrast, this study allows for gradual viewpoint adjustments based on hand movement, providing a more intuitive control experience. Second, whereas Zhang et al. (2017) [15] implemented a two-hand gesture-based viewpoint control system, the proposed method enables single-handed operation, allowing operators to adjust their viewpoint while using the other hand for object manipulation. This makes the system more suitable for teleoperation tasks where precise control of both viewpoint and end effector is required.

Despite these advantages, the system has certain limitations that require further investigation. Experimental observations revealed that cybersickness was minimal during short usage sessions or at low movement speeds but increased with prolonged usage or rapid viewpoint adjustments. This suggests that sensory conflict between visual feedback and the vestibular system can intensify if movement speed exceeds a certain threshold. In particular, repeated rapid rotational movements were found to increase the likelihood of cybersickness, which aligns with previous studies on VR locomotion. To address this issue, future research should explore methods to optimize movement speed thresholds or dynamically adjust movement sensitivity based on user motion patterns.

Additionally, this study was conducted in a controlled environment, and its performance in real-world telemanipulation or complex work environments has yet to be validated. In logistics operations, for example, various obstacles may be present, and further evaluation is needed to determine whether the proposed locomotion method effectively enhances operator visibility in such settings.

Future research will focus on enabling simultaneous control of translation and rotation with a single hand, as well as implementing motion speed constraints to mitigate cybersickness. Furthermore, additional experiments will be conducted to evaluate the impact of the proposed method on work efficiency and operator fatigue in real-world telemanipulation scenarios. These efforts will contribute to further validating the system’s practical applicability and expanding its potential use in logistics and remote operation environments.

## 5. Conclusions

This study introduced a single-hand gesture-based VR locomotion technique designed to simplify viewpoint control without requiring external devices beyond the HMD. The proposed system demonstrated its effectiveness through its ability to enable precise and continuous viewpoint adjustments using three distinct modes—translation, rotation, and fixed. By leveraging an MLP-based gesture recognition model, the system achieved robust classification performance, ensuring reliable operation across varied telemanipulation tasks.

The primary contribution of this research lies in its ability to integrate gesture-based interaction into a unified locomotion system, offering single-hand control that frees the other hand for object manipulation. This distinguishes the method from existing techniques, which often rely on dual-hand input or discrete teleportation. Additionally, the system’s hardware simplicity and seamless operation make it particularly suitable for logistics and other task-oriented virtual environments.

While the system demonstrated high accuracy and usability, findings indicated that extended use or rapid movement may increase the likelihood of cybersickness. These observations highlight the need for further refinement of motion sensitivity and system optimization to enhance long-term comfort.

Future research will focus on validating the system in complex real-world environments and exploring novel gesture designs to enhance control flexibility. Through these developments, the proposed approach has the potential to become a practical tool for improving operator efficiency and precision in virtual environments.

## Figures and Tables

**Table 1:** Distribution of gesture samples extracted from a continuous video and split into training and validation sets (8:2 ratio).

	Fist	Pointing	Unknown	Sum
Train set	1076	1436	939	3451
Validation set	268	359	234	861
Sum	1344	1795	1173	4312

## Data Availability

The data used to support the findings of this study are available from the corresponding author upon request.

## Supplementary Materials

The following supporting information can be downloaded at https://www.mdpi.com/article/10.3390/s25041181/s1, Video S1: Viewpoint adjustment using the proposed method for enhanced visibility of the target object.

## Abbreviations

The following abbreviations are used in this manuscript:

JIT	Just-in-Time
VR	Virtual reality
IMU	Inertial Measurement Unit
HMD	Head-mounted display
EMG	Electromyography
MLP	Multi-layer perceptron
ReLU	Rectified Linear Unit
